# Haptic Manipulation of 3D Scans for Geometric Feature Enhancement

**DOI:** 10.3390/s21082716

**Published:** 2021-04-12

**Authors:** Sri Harsha Turlapati, Dino Accoto, Domenico Campolo

**Affiliations:** School of Mechanical and Aerospace Engineering, Nanyang Technological University, Singapore 639798, Singapore; turl0001@e.ntu.edu.sg

**Keywords:** human-computer interaction, manufacturing/assembly, virtual environment modelling, active perception, multimodal sensor fusion

## Abstract

Localisation of geometric features like holes, edges, slots, etc. is vital to robotic planning in industrial automation settings. Low-cost 3D scanners are crucial in terms of improving accessibility, but pose a practical challenge to feature localisation because of poorer resolution and consequently affect robotic planning. In this work, we address the possibility of enhancing the quality of a 3D scan by a manual ’touch-up’ of task-relevant features, to ensure their automatic detection prior to automation. We propose a framework whereby the operator (i) has access to both the actual work-piece and its 3D scan; (ii) evaluates the missing salient features from the scan; (iii) uses a haptic stylus to physically interact with the actual work-piece, around such specific features; (iv) interactively updates the scan using the position and force information from the haptic stylus. The contribution of this work is the use of haptic mismatch for geometric update. Specifically, the geometry from the 3D scan is used to predict haptic feedback at a point on the work-piece surface. The haptic mismatch is derived as a measure of error between this prediction and the real interaction forces from physical contact at that point on the work-piece. The geometric update is driven until the haptic mismatch is minimised. Convergence of the proposed algorithm is first numerically verified on an analytical surface with simulated physical interaction. Error analysis of the surface position and orientations were also plotted. Experiments were conducted using a motion capture system providing sub-mm accuracy in position and a 6 axis F/T sensor. Missing features are successfully detected after the update of the scan using the proposed method in an experiment.

## 1. Introduction

**Figure 1:**
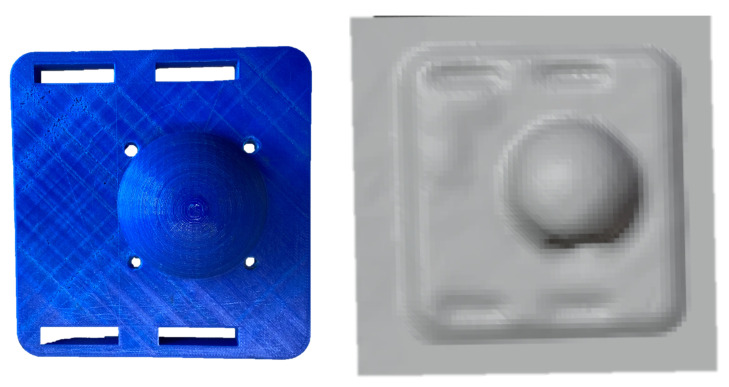
Inexpensive 3D vision systems are vulnerable to various scanning factors like ambient light, texture of the surface, etc. which affect quality of scan [5] resulting in missing features in the 3D scan of an object.

Manufacturing automation makes use of essential geometric features in object reasoning for robot planning. Features are defined as a combination of a group of relative geometric entities like a face, edge, loop or a vertex [1]. Examples of features include holes, grooves, slots, walls, etc. Ultimately, more complicated features are composed of such simpler features and are vital in robot path planning. Traditionally, localising such features is done via CAD. In applications like re-manufacturing of damaged parts, where no CAD model of the work-piece may be available, 3D computer vision becomes essential for distinguishing the defective regions [2], localizing salient features [3,4], etc. before robotised manufacturing or re-manufacturing can take place. Poorer resolution in 3D scans produced by inexpensive 3D vision systems further highlights the challenge in making robotic automation commonplace in industrial applications. Moreover, localizing features becomes all the more important when robots need to physically interact with the work-piece, i.e., for tasks such as assembly where haptic feedback is important for control. In this work, we study the haptic geometric refinement of features like holes, edges, slots (as shown in Figure 1) in models produced by inexpensive 3D scanners.

Current practice makes use of a large spectrum of 3D vision technologies to scan work-pieces to derive 3D kinematic models for robot motion planning. The availability of low-cost 3D vision systems encourages the use of robots in less-structured environments, e.g., workshops. For example, robotic manipulators nowadays are equipped with both position sensing and depth sensing along with torque sensing. Although the position sensing is within the *mm* scale accuracy, the depth sensing is slightly rougher. However, the proposed framework allows the use of fine torque sensing also available on-board to improve the quality of depth sensing by refining missing features. One of the challenges, however, is represented by the typically poorer resolution of such low-cost 3D vision systems, compared with higher-end alternatives. Although high-quality portable scanners [6] overcome these challenges using a variety of techniques [7], solutions to improve inexpensive and portable scanning would open up opportunities for small and medium enterprises (SMEs) to make use of cost-effective robotic systems. An additional constraint apart from the high cost of high-quality scanners is that they require in-situ scanning, i.e., the work-piece often has to be taken into another facility for scanning [8]. We propose to bridge these gaps by providing a flexible framework capable of refining select features interactively, to enable their localisation for robotic planning later.

The proposed framework has the following workflow where an operator (i) has access to both the actual work-piece and its 3D scan; (ii) evaluates which salient features are missing from the scan; (iii) uses a *haptic stylus* (i.e., a device capable of measuring 3D positions as well as 3D interaction forces at its end-tip, as detailed later) to physically interact with the actual work-piece, around such specific features; (iv) updates the geometry of the scan in *real time* using the position and force information from the haptic stylus.

This work, however, will only focus on the latter points, i.e., on the way the initial scan is updated, for the purpose of enhancing salient features based on data derived from the physical interaction with work-piece via the haptic stylus. More specifically, in this paper we will *not* address the subsequent robotic motion planning, including calibration problems between robot coordinates, work-piece coordinates and 3D scan coordinates. Furthermore, we shall assume that the operator, based on his/her experience, knows which salient features are needed for a specific task. In this sense, we will not deal with exploration strategies which vary from operator to operator and are task-dependent.

As explained in the following sections, we use a discrete mesh as a numerical estimation of the actual work-piece surface. Initialized with a 3D scan, this mesh is continuously updated based on the physical interaction between the work-piece and the haptic stylus held by an operator. The novelty of our work is that the geometric update of the mesh is based on the *haptic mismatch* between the *actual force*, as sensed by the haptic stylus from the interaction with the actual work-piece, and the *expected force*, which is predicted based on the geometry of the surface and current position of the stylus. It should also be noted that the information used to update the geometry of the surface in this process not only makes use of the 3D position of the stylus end-tip, as typically done in digitization techniques, but also of the 3D forces (magnitude and direction) arising from the actual interaction, hence the name ’haptic’ stylus. In particular, the mesh (from the 3D scan) is updated only in presence of *haptic mismatch*. The 3D position of the stylus determines where to update the mesh while the 3D forces are used to determine the direction and magnitude of the update. This is in contrast with the other approaches in haptic digitization. Specifically, most methods use the knowledge that there was physical contact at a location as a way to *record* this location as part of the surface model. This will be detailed in the next section. Some challenges with these methods include thresholding the sensed contact to avoid outlier points, filtering the *recorded* points and fitting a surface on them. The computational demand in implementing these methods in real time is yet another challenge. The proposed framework overcomes these challenges by using the *forward* prediction of haptic feedback obtained from the initial 3D scan and then uses the backward haptic feedback to refine it, in real time.

The rest of the paper is organized as follows. In Section 2, we present various scanning and digitization methods as a background for this work and highlight our novelty. In Section 3 we introduce the proposed framework and mathematically define haptic mismatch. Section 4 simulates haptic mismatch through numerical simulations of the proposed framework on smooth differentiable surfaces to validate the geometric convergence of the different features being refined. Finally Section 5 details the experimental refinement of missing features (like holes, edges, slots) from 3D scans of objects, followed by the conclusions and future scope of this work.

## 2. Background

In this section we review different non-contact and contact based scanning technologies and the trade-offs between them. The challenges in using high end scanners in terms of cost, additional expert skill requirement, lack of mobility and limited scanning space are identified as gaps our framework aims to address. Various available 3D model formats for representing the surfaces are presented. Haptic based scanning methods are surveyed to discuss the framework’s relevance in robotics research. Finally, haptic sculpting literature and its relevance to our work is studied to highlight the novelty in our approach.

### 2.1. Non-Contact and Contact Based Scanning Technologies

Manufacturing industries have employed non-contact based 3D scanning technologies such as laser triangulation, structured light, time-of-flight and photogrammetry [9] in part inspection and reverse engineering applications [10]. Laser triangulation offers high resolution and accuracy but suffers from sensitivity to surface properties [11] (like roughness, reflectivity, transparency, etc). Similarly, structured light scanners are dependent on appropriate lighting conditions [12], time of flight sensors cannot detect concavities in shape and photogrammetry requires high computational power [13]. Optical 3D coordinate measure machines of the manufacturer ATOS GOM [8] and its competitors use a wide variety of scanning technologies including structured light scanning with high accuracy results and often are able to capture fine features too, but prove very expensive for setting up and require expert skill to operate.

Different non-contact sensors capture 3D information and use a variety of output formats. Popular models to visualize the 3D geometry include point clouds, polygonal meshes, Non-uniform rational basis spline (NURBS) surface models, etc. Interconversion between these models is well established. In this work we use Occipital’s structure sensor [14] which comprises of an IR structured light projector and a camera that captures the reflected pattern. A set of algorithms compute the 3D scene information over several iterations by tracking the relative position from which a frame of 3D information was recorded over time. A triangular mesh is produced, like the one rendered in Figure 1. Triangular meshes are widely used in 3D vision and a recent approach to building a mesh from a single image was presented in [15] where the depth map of an image was used to *wrap* a plain mesh for synthesising novel views. The proposed work is an analogue to this method but is driven by forces from physical interaction instead of visual features. Specifically, the *wrapping* of a mesh to conform to absent geometric features using haptic feedback is the haptic analogue we present.

Among contact based high resolution scanning methods, one of the early inventions is the Atomic Force Microscopy (AFM) method [16]. This method is worth mentioning since our work is fundamentally similar to this. Although it allows for measurements at the *μm – mm* level and understanding material properties like magnetism, elasticity, etc., one of the main drawbacks is the limited mobility and the special requirements like vacuum environments for operation. Coordinate measuring machines (CMMs) have also used physical contact to improve coordinate metrology [17]. Other contact based scanning methods like Tactile probing [18] for manufacturing applications like part inspection [19] and reverse engineering [20] by linking vision to touch are also not new. However, Refs. [17,18,19,20] specifically require CAD and are primarily post-processing methods limited to verifying part geometry after manufacturing. Our framework on the other hand, is a pre-manufacturing process for refining 3D scans with finer features omitted during scanning.

### 2.2. Haptic Digitization

Digitization is the process of reverse engineering virtual models of actual objects. Conventional contact based scanning methods for digitization largely targeted at part inspection were discussed in the previous subsection. However, *haptic digitization*, i.e., the use of active touch to reconstruct the shape of an object for perception of size and position is also an active field of research. Haptic perception caused when people move their hands over an object to decipher smoothness, roughness, curvature, etc. [21] is attributed to *Haptic exploration*. Haptic exploration may be used for feature detection, i.e., of bumps, cracks, ridges, etc. Early approaches to this problem proposed incremental updates of a probabilistic map of the environment for a known smooth surface [22] and unknown objects [23]. This is also known as *Haptic SLAM*. Similar implementations using particle filters [24] have demonstrated accurate reconstruction of a Rubik’s cube and [25] presented visual-tactile fusion for object recognition with fingered hands. Object classification through blind tactile exploration in the presence of sensory noise was also shown in [26]. Some more noteworthy robotics applications of haptic digitization are surface patch reconstruction [27], localization of objects [28], improved grasp planning [29]. Interesting approaches to digitization by means of fusing visual information with touch information have also been done for grasp planning applications as listed next. Ilonen et al. [30] used visual information and tactile information together to help extract shape information and reduce uncertainty, however with the assumption of symmetric objects. An implicit surface was modelled as a Gaussian process in [31,32,33,34] and further refined using touch information. Shape priors were extracted from visual images and then corrected using tactile data for 3D shape perception [35].

We next study various techniques in sculpting, which is the step that follows digitization of an object.

### 2.3. Haptic Sculpting

Our work draws inspiration from many sculpting techniques already in use for computer aided manufacturing applications. Haptic sculpting constitutes subtracting of materials from digital objects [36] to modify their shape. Haptic sculpting has been used for product prototyping [37], redesigning a reverse engineered product [38], repairing incomplete measured data [39] and collaborative sculpting [40]. To aid with parametric representations in CAD like NURBS and Basis spline (B-Spline), specialized haptic sculpting methods were also devised to perform sculpting of rigid [41] and deformable objects [42] and finally manufacturing planning [43]. Out of all these approaches, the work presented in [44] is the closest to our work. Interestingly they use haptic sculpting for the more fundamental process of digitization by initializing the digital object with a virtual clay. This virtual clay is sculpted through volume subtraction virtually using just position data from the haptic interface. Our work uses *actual* haptic feedback, i.e., the force readings recorded during physical interaction with the object and differs from them fundamentally in this sense.

### 2.4. Theoretical Comparison and Challenges across Touch Based Scanning Methods

Most of these surveyed approaches focus on extracting the shape of an object or a surface using vision or touch or both. The common applications across them were either (i) object shape estimation/reconstruction/repair or (ii) localization of object by way of reconstruction or acquiring samples of surface points. One commonality among these methods is the use of a sensorized stylus or a tactile array capable of providing end-tip position and contact (on/off) information to perform point-wise virtual mapping using touch information—a binary view of physical touch. Spatial location $x_{i}$ is usually associated with a *target value* $y_{i}$ which is indicative of whether the point is in the interior ($y_{i}<0$), on ($y_{i}=0$) or in the exterior ($y_{i}>0$) of the surface in question. A commonly used model in these approaches [31,32,33,34,45] is a gaussian process implicit surface (GPIS). It would seem an appropriate choice of a surface model since it readily incorporates the uncertainty involved in measurements. There are other models also used like the point cloud in [29,30], occupancy grids/voxels [24,44] and surface polynomials in [27,46,47]. However, the touch information collected in all these approaches is binary—evaluating whether or not contact has occurred without using the forces sensed at the point of contact and the vital information of surface normal that it encodes. This is one major gap we address in our proposed framework—we use both the magnitude and directional information provided by the forces at the point of contact to extract/refine the perceived shape of the surface. A few works [28,48] do use the forces sensed at the contact point for localisation purposes, but not for refining the perceived shape of an object. Moreover, their method relies on prior knowledge of the object geometry before localisation is possible and cannot be used with unknown objects as is required in remanufacturing applications.

From a computational standpoint, most of the approaches collect touch information as a tuple $\left(x_{i},y_{i}\right)$, where the $y_{i}$ is obtained by evaluating whether or not contact occurred and the GPIS or any model they use is retrained/regressed to capture the newly perceived geometry. For fully automated scenarios while these approaches may be useful, in cases that require interactive and intuitive interfacing with a human operator in the loop (for instance in manufacturing and remanufacturing settings) such retraining of models present a computational challenge. Moreover, most of these methods use tactile arrays for collecting touch information, which are not ideal for abrasive or rough surfaces typically encountered in remanufacturing settings. In this work we bridge these aforementioned gaps by proposing a novel framework that allows for interactive haptic sculpting of a scanned surface by using both the magnitude and directional information from the sensed physical contact. The model used is a 3D polygonal mesh, which is refined/morphed explicitly (as opposed to an implicit parameter that encodes the surface information) using differential equations. This is in contrast to the retraining step that is the state of the art currently [45]. By designing the differential equations with a visco-elastic form, we ensure the refinement is intuitive and the mesh deforms in a *clay-like* manner to *actual* forces exerted by the human operator on the real object using the haptic stylus.

These works in touch based scanning, haptic digitization, *Haptic SLAM* and haptic sculpting are very similar to the proposed framework in this paper. However, our work differs from them in some fundamental ways. Our key contribution is the idea to use the geometry available from the 3D scan to compute an *expected* haptic feedback at a point and compare it with *actual* haptic feedback to derive a *haptic mismatch*. This in turn is used to drive the update of the 3D scan. In terms of how the virtual model is morphed, most of the surveyed methods assume voxel-based representations of objects or GPIS and involve volume subtraction or updating voxel occupancies and gaussian process regression for mapping the surface of an object. Our methodology morphs the 3D model locally at the surface directly.

In the following section, we provide an overview of the proposed framework following which we mathematically formulate it. We also formalise the notion of *haptic mismatch* and define the two ways in which the update of the 3D scan at a local point occurs, i.e., position and orientation corrections.

## 3. Proposed Framework

**Figure 2:**
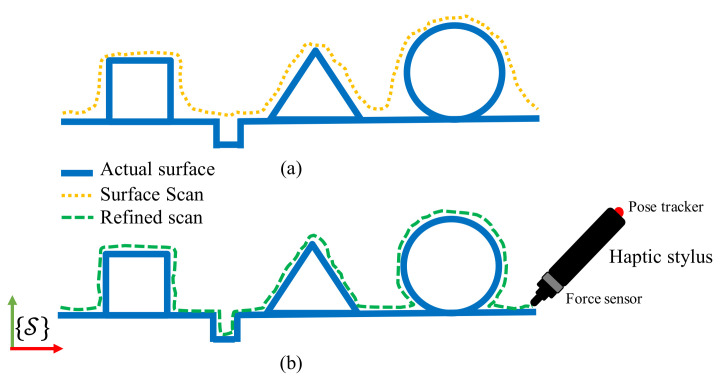
(**a**) 2D sketch depicting the geometric discrepancies between an actual surface and its scan which we propose to refine/correct using (**b**) a haptic stylus through physical interaction with the actual surface. Firstly, we use the scan’s geometry to predict the virtual interaction with the scan. We define a haptic mismatch which is the difference between the prediction and the sensation of interaction with the actual surface. The geometric discrepancies like the edges of the square and the slot are corrected using haptic mismatch.

We propose a framework whereby a human operator, after a visual inspection of the initial scan, may proceed to a manual refinement of the scan by means of a haptic stylus used to physically interact with the actual, rigid surface (Figure 2b). Consider a rigid object and a scan of its surface as sketched in Figure 2a. The scanned surface very often will fail at capturing certain details, e.g., holes, sharp edges etc. The force arising from the physical interaction with the actual surface will be used as input signal to a filter, which will adapt the triangular mesh derived from the the scan locally, i.e., in the area of physical interaction and with a magnitude proportional to the force itself.

To record the haptic feedback, we use a haptic stylus as shown in Figure 2b. The haptic stylus will be equipped with a force sensor that provides the haptic feedback of the *actual* interaction with a surface. Additionally, the position and orientation of the stylus are also to be sampled for real-time rendering purposes.

Upon surface-tool interaction by the haptic stylus with a real surface an *actual* haptic feedback is recorded in terms of forces and torques. Concurrently, the *internal* model *predicts* the *expected* haptic feedback. The internal model is a haptic rendering of the mesh structure obtained from the 3D scan, i.e., the geometry of the 3D scan is used to compute a prediction of the force feedback to be expected when a tool comes in contact with a surface point. This prediction is then compared with the *actual* haptic feedback to produce a *haptic mismatch*. The *haptic mismatch* coupled with the *haptic update* rules of the framework correct the surface scan geometry in terms of position and orientation of individual surface points to reflect the real surface’s geometry accurately.

To model surfaces in 3D we use triangular meshes because they allow to work with a locality in the model while keeping the rest of the regions of the mesh intact. This allows for efficient implementations. To mathematically describe the mesh obtained from the scan of the real surface in a reference coordinate system, we define state variables. These variables will be used to define the interaction function used to calculate the *expected* haptic feedback, given a position of the haptic stylus. The two types of *haptic mismatch* for position and orientation correction of a surface point in the scan are presented with qualitative case studies. Briefly, these cases are meant to understand the positional and orientation haptic mismatch for different errors in the surface scan w.r.t the actual surface. For instance, based on whether or not the haptic stylus is penetrating the 3D mesh, an *expected* haptic feedback—both in terms of magnitude and direction may be computed. This may or may not be non-zero and both these cases may correspond with a zero or non-zero *actual* haptic feedback sensed. Studying these cases will help understand the working of the proposed framework. Consequently, the mathematical rules to correct the haptic mismatch are summarized and a numerical validation of these rules for one surface point is presented. These rules constitute the aforementioned *haptic update*. Finally, error metrics are defined and a numerical simulation of the haptic update is carried out in the 3D case to quantitatively characterize the framework’s performance.

### 3.1. State Variables

**Figure 3:**
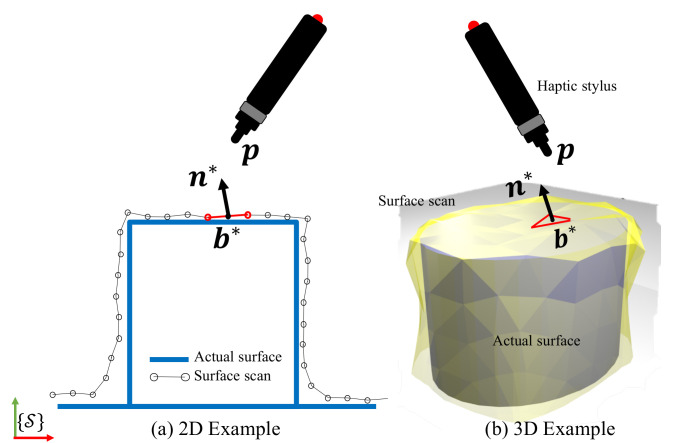
Definition of state variables on surface scan and haptic stylus.

Figure 3a describes the virtual surface scan as a piece-wise linear approximation of the actual surface geometry. Since the scan of any surface is a discretised *estimate* of the surface geometry, we denote the state variables related to the scan with the superscript ${}^{*}$ and those related to the actual surface without. The state variables are defined in the space frame {S}. Considering the haptic stylus’s pen-tip at position $p\in R^{3}$, the closest point to the pen-tip on the mesh is denoted by $b^{*}\in R^{3}$, the barycenter/centroid of the closest face. Similarly, the orientation information of the geometry is captured by the surface normal variable $n^{*}\in R^{3}$. These state variables will be adapted based on sensory inputs as explained in the next subsection.

The basic idea of the framework is to adapt ($b^{*}$, $n^{*}$) based on haptic mismatch. Only when the haptic stylus is in contact with the surface, i.e., non-negligible interaction forces are recorded. The framework aims to adapt $n^{*}$ and $b^{*}$ such that the respective face is on the tangent plane to the surface at the point of interaction.

### 3.2. Calculating *Expected* Haptic Feedback

**Figure 4:**
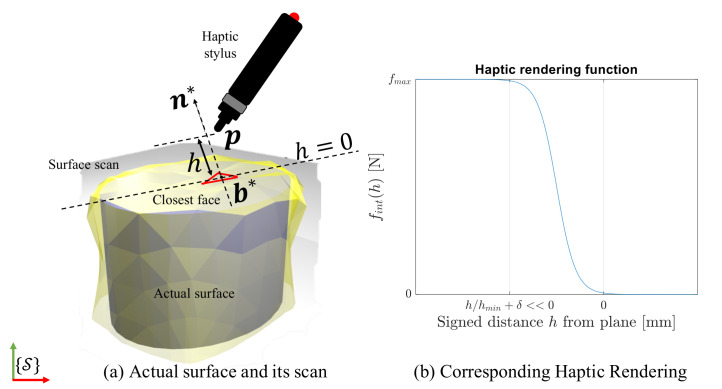
Calculating expected haptic feedback using haptic stylus position and scan geometry.

The mesh represents the actual surface to the best of the sensor’s accuracy and as such, it will be used to predict interaction. In other words, if the haptic stylus was to penetrate a given face of the mesh, we would expect an interaction force perpendicular to the face normal (assuming no friction with the actual surface). In order to calculate the *expected* force (haptic feedback), it is useful to define a signed distance *h* as:

(1) $$h=\left(p-b^{*}\right)\cdot n^{*}$$

where · is the Euclidean dot product. This is shown in Figure 4a. Given the haptic stylus position p, we define the *expected* haptic feedback $F^{*}$ using the haptic rendering function $f_{int}\left(h\right)$ and the normal at the point in the scan as:

(2) $$F^{*}=f_{int}\left(h\right)n^{*}$$

The nature of $f_{int}\left(h\right)$ is depicted in Figure 4b and in this work, is defined as an adjustable sigmoid function:

(3) $$f_{int}\left(h\right)=\frac{f_{max}}{\left(1+e^{h/h_{min}+\delta }\right)}$$

where $h_{min}$ is the depth tolerance—the minimum distance from the surface at which, interaction forces are said to exist between the point of interaction and the surface and $f_{max}$—the maximum force the model simulates. δ controls the point of inflection of the sigmoid function and may be chosen such that the interaction force is strictly zero for positive *h*, i.e., when there is no penetration. The interaction force function $f_{int}$ is meant to be a smooth transition between a zero and non-zero contact over a range of penetration depths.

The framework’s performance is sensitive to the interaction force function along the domain—signed distance *h*. The lower the depth tolerance, the stiffer the function, i.e., for lower displacements, a higher contact force is estimated. Furthermore, the interaction force function allows a choice over the range of forces (from 0 to $f_{max}$) to be expected in an application and a smooth variation over that range.

### 3.3. Haptic Mismatch Correction

**Figure 5:**
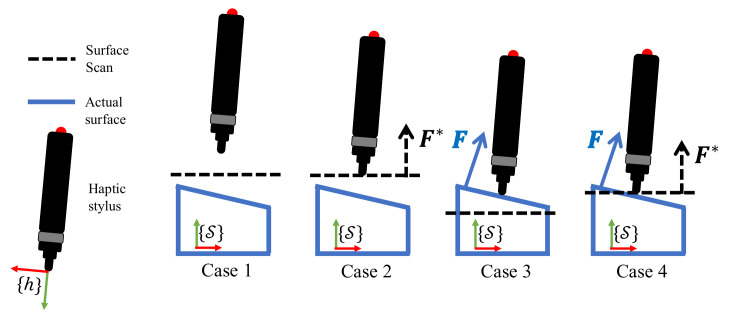
Cases of correcting *Haptic mismatch*: the situations where the haptic stylus is interacting with the mesh, the surface, both or neither and therefore computes a zero/non-zero *expected* and *actual* haptic feedback.

We define *haptic mismatch* as the difference between the actual interaction and the virtual interaction calculated w.r.t the mesh. The most common case while evaluating a *haptic mismatch* is when the haptic stylus is hovering in space and is neither in contact with the mesh, nor the actual object. We label this as Case 1 as shown in Figure 5 where the actual haptic feedback F and the expected haptic feedback $F^{*}$ are both absent, i.e., null in magnitude. When the haptic stylus is only in contact with the mesh and not the surface, a non-zero *expected* haptic feedback $F^{*}$ is calculated. On the other hand, when in contact with only the surface and not the mesh, a non-zero *actual* haptic feedback F is recorded. Cases 2 and 3 describe these. Finally when the haptic stylus is in contact with both the mesh as well as the actual surface, the framework evaluates non-zero $F^{*}$ and F as seen in Case 4. Note that the *actual* haptic feedback will be sensed in the haptic stylus frame {h} and must be converted and expressed in space frame {S} when computing haptic mismatch. To match the scan geometrically with the actual surface, we design the framework to align all the face normals of the scan to the actual surface normals at their respective positions. The haptic mismatch is said to be corrected fully if the *expected* haptic feedback at every discrete point on the scan matches the *actual* haptic feedback at that same location on the actual surface.

Mathematically, a face in a mesh is fully described by its barycenter (position information) and its face normal (orientation information). These are the two vector variables that will be adapted to correct the haptic mismatch using the *expected* and *actual* haptic feedback. The update of the barycenter is defined as the *position correction* and the update of the face normal as the *orientation correction*.

#### 3.3.1. Linear Haptic Mismatch:Position Correction

The purpose of the position correction of the face barycenter on the mesh is to ensure that it lies on the plane described by the actual point of contact and the surface normal there. To this end we define the linear error in forces by:

(4) $$\epsilon_{L}=F-F^{*}$$

The correction of linear haptic mismatch is essentially a linear translation of the face of interest on the scan along its normal direction $n^{*}$. The idea is to preserve the normal information suggested by the scan until new information from the actual surface is perceived through the actual haptic feedback F. Once there is an actual interaction force encountered by the F/T sensor, F would increase to a non-zero value, thus *moving* the face of interest along its normal until $|\epsilon_{L}|=0$. This would only happen when the barycentre $b^{*}$ of the scan face is on the actual surface.

In the case studies in Figure 5, the point of contact may be deciphered from the haptic stylus position while the surface normal may be obtained from the forces sensed. Since $|\epsilon_{L}|=0$ in Case 1 always, there will be no position correction when the haptic stylus is not in contact with both the mesh and the surface. In Cases 2 and 3, the correction of haptic mismatch would entail purely position correction.

#### 3.3.2. Rotational Haptic Mismatch:Orientation Correction

Once the barycenter of the face (from the mesh) lies on the actual surface, the next step is to ensure the face normal matches with the surface normal. We define a rotational error in forces by:

(5) $$\epsilon_{R}=F^{*}\times F$$

The cross product rule may be viewed as analogous to inducing a moment on the face to rotate it until rotational equilibrium is reached. This would occur when both the *expected* haptic feedback and the *actual* haptic feedback match in terms of their vector direction. In other words, the unit vector along F describes the actual surface normal at the point of contact. The orientation correction is performed by rotating $n^{*}$ to align with the unit vector along F once the linear *haptic mismatch* is corrected through the position correction. Note that the interaction force at any point on the surface is along the surface normal there. Hence, the cross product of $F^{*}$ and F would be a zero vector if the two faces were aligned. This cross product thus defines the direction about which the $n^{*}$ needs to rotate to align with the surface normal, described by the unit vector along F. Finally, a triple cross product with $n^{*}$ serves the purpose of deriving the rotational *haptic update*.

The overall correction of *haptic mismatch* may be summarised as simply a positional correction for Cases 2 and 3 and a positional and orientation correction for Case 4. This may also be noticed from Figure 5 which shows that $\epsilon_{R}$ may be non-zero only in the last case.

### 3.4. Haptic Update

A summary of the *haptic update* rules qualitatively described in the previous subsections is presented next. The state variables are (i) the barycentre $b^{*}$ i.e., centroid of the closest face on the scan to the haptic stylus and (ii) the face normal $n^{*}$. The haptic mismatch is carried out in two parts as described before:linear and rotational. The overall correction of *haptic mismatch* is done by solving the following differential equations defined on the state variables:

(6) $$\mathrm{Position}\mathrm{correction}:\frac{d}{dt}b^{*}=\alpha \left(\left(F-F^{*}\right)\cdot n^{*}\right)n^{*}\left(0\right)$$

(7) $$\mathrm{Orientation}\mathrm{correction}:\frac{d}{dt}n^{*}=\beta \left(F^{*}\times F\right)\times n^{*}$$

Together, Equations (6) and (7) constitute the *face dynamics* of the scan. The variables in these update rules are listed out in Table 1. Typically, the scan of a surface is a close enough approximation of the actual surface and the refinement of the scan should be such that it does not morph too much too abruptly. To ensure this behaviour, we introduce admittance coefficients α and β which control the spatial evolution of the face barycentre $b^{*}$ and normal $n^{*}$. These also contribute to ensuring stability of the *haptic update*.

## 4. Numerical Simulation of Haptic Update

In this section, to numerically validate the proposed framework, we define the haptic stylus dynamics, i.e., dynamical behaviour of the haptic stylus position p(t). The *haptic stylus dynamics* combined with *face dynamics* are defined as *coupled dynamics* of the *haptic update*. The set of differential equations from the coupled dynamics are solved using standard numerical solvers and error functions are evaluated over the results to quantify the performance of the framework. The application of the framework in the 3D case is also presented with numerical simulation results for the same.

### 4.1. Coupled Dynamics

For simulation purposes we define the constraints on the haptic stylus motion, p(t) to perform the contact task. Robots are often required to apply a desired force on surfaces in tooling tasks and moreover need controllers to command their position and forces. We define a few intuitive guiding forces to mimic the time-profile of haptic stylus position similarly. The first guiding force we use is $F^{el}$—an elastic force that guides the haptic stylus to a point of interest on the scan i.e., $b^{*}\left(t\right)$ since the closest point on the scan is of importance. Secondly, a desired contact force that the haptic stylus should apply on the surface is defined as $F^{con}$. These are defined in relation to the state variables as:

(8) $$F^{el}=K\left(b^{*}-p\right)$$

(9) $$F^{con}=-F_{c}\;n^{*}$$

The forces influence the *haptic update* simulation resulting in the haptic stylus (described by its position p(t)) always being attracted to the closest face on the scan (whose location is specified by $b^{*}\left(t\right)$) while attempting to apply a desired contact force $F^{con}$. Combined with the viscous coefficient γ, the update rule for p, is defined in Equation (10).

(10) $$\mathrm{Stylus}\mathrm{position}\mathrm{update}:\frac{d}{dt}p=\gamma \left(F+F^{el}+F^{con}\right)$$

Note that the reaction force of the actual surface on the haptic stylus denoted by F also has an influence on the haptic stylus dynamics. In other words, the equilibrium point for the haptic stylus for some point of contact will be the resultant of the three forces F, $F^{el}$ and $F^{con}$. In real experiments, p may be human driven or robot driven—we leave this flexible to cater to different applications.

The coupled dynamics are denoted by:

(11) $$\frac{d}{dt}Y=\Phi \left(Y,F\left(t\right)\right)$$

where $Y={\left[p\;b^{*}\;n^{*}\right]}^{T}$, the compound state vector. This compound state vector will be evolved based on the individual update rules in Equations (6), (7) and (10).

### 4.2. Simulation and Error at Equilibrium

**Figure 6:**
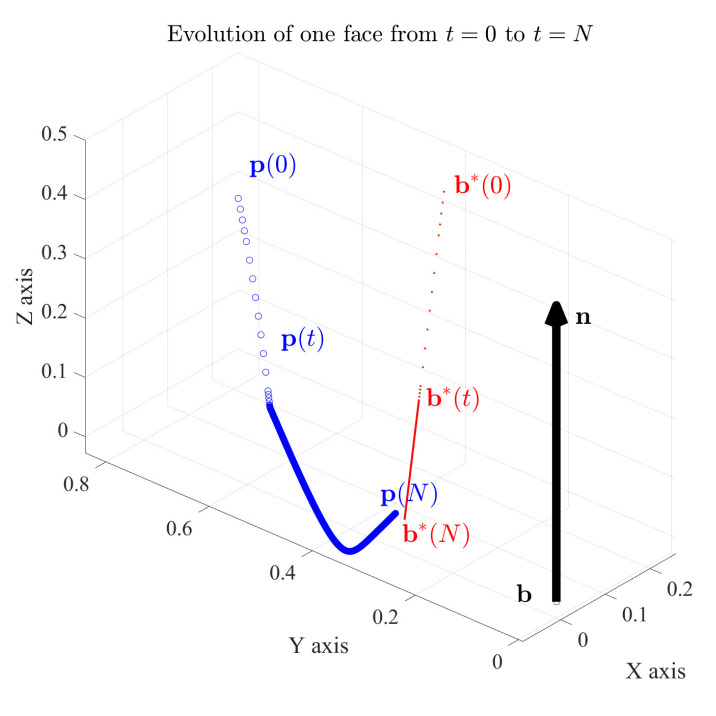
State update for one face with barycenter $b^{*}$ and face normal $n^{*}$.

The state variables ($b^{*}$, $n^{*}$) were initialized to random vectors in $R^{3}$ with $h_{min}=0.5$ mm and δ=5. The solutions to differential Equations (6), (7) and (10) using the *medium order method* with *ode*45 solver in MATLAB are shown in Figure 6 which is the outcome of *haptic update* for one surface point. We define dynamic equilibrium for the state variables when they are unchanging. In practice, we need only run the *ode45* solver over the differential equations for a finite time (t=N) for dynamic equilibrium. N is set heuristically. Note that theoretically, at dynamic equilibrium (t=N) following *haptic update*, i.e., $b^{*}$ may or may not coincide with b—they may be observed as far apart in Figure 6. However, the plane defined by b and n will be the same as the plane defined by $b^{*}\left(t=N\right)$ and $n^{*}\left(t=N\right)$. We define b and n as the variable analogues for the actual surface of the *estimated* state variables ($b^{*}$, $n^{*}$) for simulation purposes.

**Figure 7:**
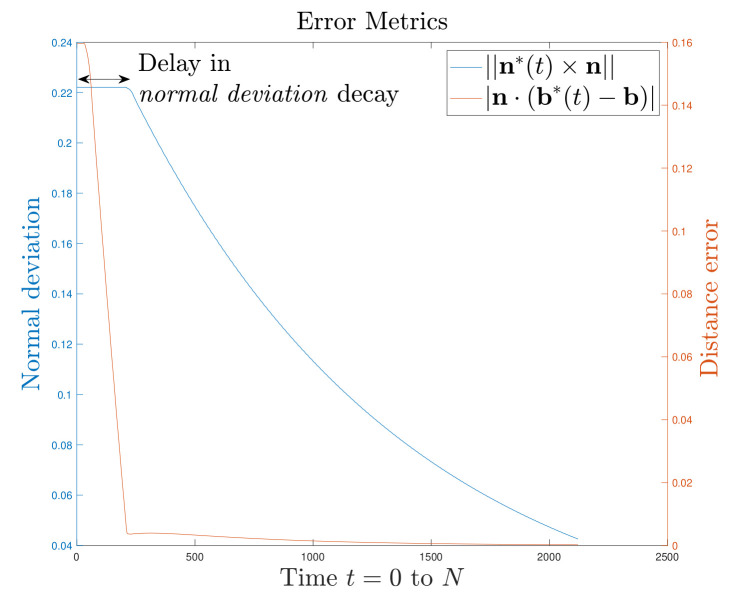
Evolution of error metrics over time for one face.

We characterize error metrics evaluating the scan (as it gets refined) with respect to the actual surface. The error metrics are defined as deviations of $b^{*}\left(t\right)$ and $n^{*}\left(t\right)$ from b and n. The normal distance between face planes, $\left||(b^{*}\left(t\right)-b)\cdot n|\right|$, and the angular deviation between face normals, $|n^{*}\left(t\right)\times n|$. We name these error metrics the *distance error* and *normal deviation* respectively. The behaviour of the framework based on these is characterized in Figure 7. From the graph it can be observed that the face first adapts to the *haptic update* rule in Equation (6) (Position correction) before beginning to respond to Equation (7) (Orientation correction). In other words, the face adapts to the normal distance between face planes along the $n^{*}\left(0\right)$ as observed by the straight trail p(0−t) in Figure 6. This is the *position correction* part of the *haptic update* and may be observed how the *distance error* is corrected first in Figure 7. The *haptic update* rule in Equation (7) for correcting the face normal deviation (Orientation correction) may be observed in how the decay in *normal deviation* is delayed in Figure 7.

### 4.3. Local Deformation of Meshes for Selective Feature Refinement

**Figure 8:**
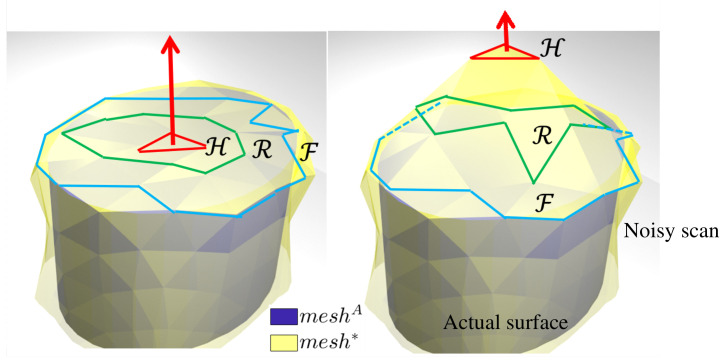
The actual surface and its scan are modelled as triangular meshes. Neighbourhood (ROI) selection for mesh deformation for a certain displacement of the handle vertex set H.

For simulation purposes, we model the surfaces as triangular meshes. The actual surface is denoted by *mesh*^A^, where ’A’ stands for *actual* and its scan mesh*. The superscript ^*^ is indicative of mesh* being an estimate of the actual surface *mesh*^A^, as shown in Figure 8. In a large mesh each face is surrounded by several other faces. The relative size/area of these faces with respect to the haptic stylus’s pen-tip size should be used to determine the region of interest in mesh* for refinement. This local implementation allows to be computationally efficient while storing large amounts of information. We use mesh morphing strategies borrowed from works in computer graphics along with the proposed *haptic update* rules to adapt neighbourhoods in a mesh using *actual haptic feedback*. Thus, the exploration performed by the haptic stylus must affect only the faces in contact with the pen-tip, with minimal disturbance caused to the rest of the mesh. Ideas on mesh morphing methodologies are referenced from [49,50] to implement such selective neighbourhood deformation in a 3D mesh.

In this work, we use the Laplacian surface editing. Given the haptic stylus position p, the closest face f* and its barycenter $b^{*}$ are identified by:

(12) $$b^{*}:=\underset{b\in mesh^{*}}{\mathrm{argmin}}{\left|p-b\right|}^{2}\;\;\;$$

For each face visited during the haptic exploration task, a Laplacian matrix is constructed for the vertices involved with the closest face f* and their surrounding 1-neighbours. The region of interest *ROI* for mesh deformation for a certain displacement of the closest face may be seen in Figure 8. It constitutes a handle region H that is defined over the vertex set of the closest face, a free region R defined over the vertex set in the surrounding neighbourhood and a fixed region F defined as the boundary vertices that round up all these vertices. Ideally the size of this neighbourhood must be determined using the area the pen-tip would cover on the mesh.

**Figure 9:**
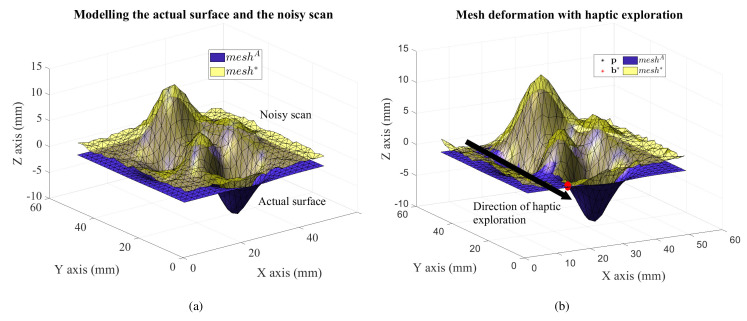
(**a**) The actual surface and its noisy scan are modelled as triangular meshes, (**b**) The red bead represents the state variable $b^{*}\left(t=N\right)$ at dynamic equilibrium when the haptic stylus’ tip p is at the point of contact (coincident with $b^{*}\left(N\right)$ here) while performing haptic exploration on a section of *mesh*^A^ in a direction parallel to the Y axis. The deformation of mesh* may be clearly observed as approaching *mesh*^A^.

To realistically model noise in mesh*, we rely on the precision specifications of the sensor we will later use in experiments to collect 3D information of a scene, i.e., the Occipital structure sensor, which on average has a precision of 1 mm [14]. So, mesh* was obtained by adding 3D Gaussian noise of 3 mm standard deviation to each vertex of *mesh*^A^ and a vertical error of 2 mm to attempt a simulation test of the framework in worse conditions than practicable. This may be seen in terms of how much smoother *mesh*^A^ is than mesh* in Figure 9a. Both the meshes were generated using the *peaks* function in *MATLAB* with the dimensions 50 mm × 50 mm × 20 mm. This produces smooth differentiable surfaces for the framework to be numerically tested on first. The objective of the framework is to carry out a *haptic update* of mesh* to minimize the *haptic mismatch* between *mesh*^A^ and mesh*.

Both the meshes are referenced in the same global coordinate system. To check whether or not a surface point on mesh* actually lies on the real surface, the haptic stylus position is set to this point. Correcting any haptic mismatch needs to be done one surface point at a time, since touch is localized point wise in haptic applications. Therefore, at any instant, we focus our analysis on one triangular face over both meshes, *mesh*^A^ and mesh*. To verify the dynamics of our framework, the *expected* and *actual* contact forces, $F^{*}$ and F which are the interaction forces w.r.t mesh* and *mesh*^A^ (in reality would be a feedback from a force sensor) respectively, are calculated using haptic rendering techniques, using the surface penetration based contact model as shown in Figure 4 and as explained in Section 3.2.

The framework is initialized to these conditions and the state variables ($b^{*}$, $n^{*}$) of the closest face to the haptic stylus are evolved using the *haptic update* rules to refine the mesh. We use the 1-neighbourhood Laplacian surface editing for mesh deformation. The mechanism of *haptic update* may be observed along the path of the red bead moving on the mesh* in Figure 9b as the mesh* is morphed to conform to the actual surface modelled by *mesh*^A^.

#### Geometric Convergence of Meshes by Minimising Haptic Mismatch

**Figure 10:**
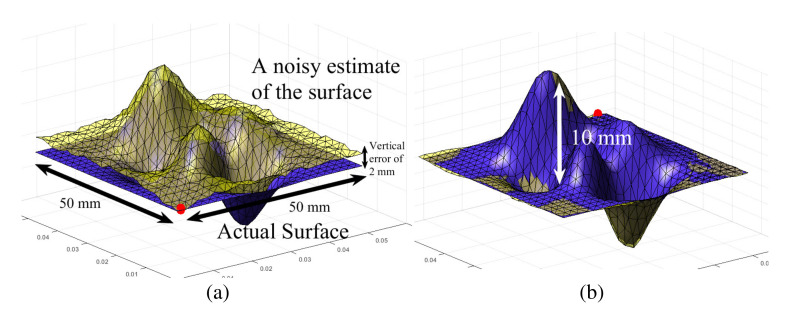
(**a**) The geometric discrepancy in estimation of the real surface is shown by the deviation of faces in *mesh*^A^ (depicted in blue) from the corresponding faces in it’s modelled 3D scan, mesh* (in yellow). (**b**) This geometric discrepancy is corrected after *Haptic update*.

Haptic exploration was performed over all the faces on mesh* with the haptic stylus dynamics described in Equation (10) and the *haptic update* rules employed to each of the faces’ state variables, i.e., $b^{*}\left(t\right)$ and $n^{*}\left(t\right)$ and the resultant mesh with near-zero haptic mismatch may be seen in Figure 10b.

**Figure 11:**
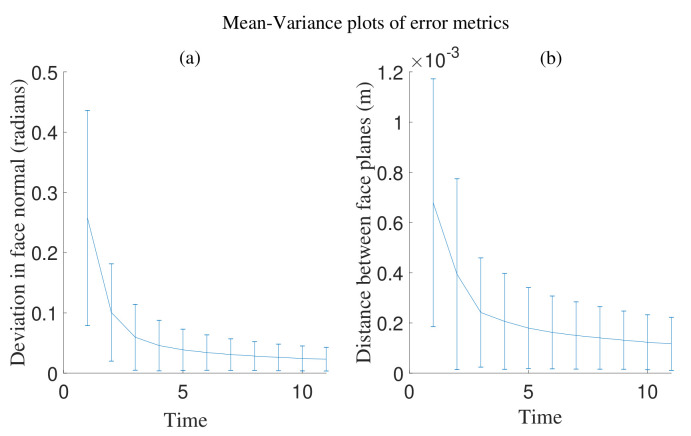
The combined error of (**a**) face normal deviation and (**b**) normal distance between face planes *mesh*^A^ and *mesh*^A^.

Quantitatively, the error metrics introduced in Section 4.2, i.e., *distance error* and *normal deviation* were evaluated on average for all the faces involved in the meshes. It was observed that they decay exponentially with the number of sweeps of haptic exploration as seen in Figure 11, validating the framework’s performance in correcting haptic mismatch through haptic exploration. From the results in Figure 11, it can be seen that, over several iterations of *haptic update* (i.e., after ten sweeps of haptic exploration) the deviation in face normals and the distance between face planes decreased consistently. We can design this process to be faster by choosing the viscous coefficients of the haptic update (α, β) wisely. Over several other experiments, it was also observed that the meshes were sensitive to relatively higher doses of noise. The face size in comparison to the 3D noise in the information that the face contains, has a significant impact on the performance of the framework. In other words, the problem is ill posed if the normal at a surface deviates too much from the face normal obtained from the scan.

## 5. Experimental Refinement of Geometric Features

**Figure 12:**
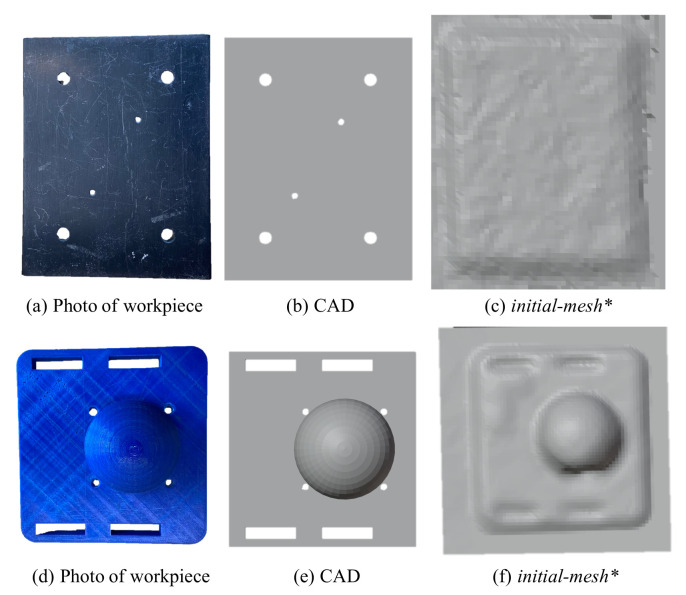
(**a**,**d**) The object of interest, (**b**,**e**) the CAD of the object with distinct features, (**c**,**f**) the initial-mesh* obtained from the 3D scan of the object with missing features such as the holes and the edges smoothed out.

In the previous section, the numerical convergence of the proposed framework was performed on smooth differentiable surfaces. To validate the framework’s efficacy on real geometric features suitable for robotic assembly, we pick objects with distinct features like holes, edges and slots (see Figure 12). It may be noted that the CAD (Figure 12b) is not usually available in remanufacturing settings, but is shown here for the purpose of highlighting the features absent in the 3D scan of the same object (Figure 12c. Although the missing features in these objects are primitive ones (holes, edges and slots), a vast majority of the geometric features in industrial applications are composed of these. To refine these missing features in the 3D scans, both position and force data from the haptic stylus were obtained as described next.

### 5.1. Data Acquisition Methods

**Figure 13:**
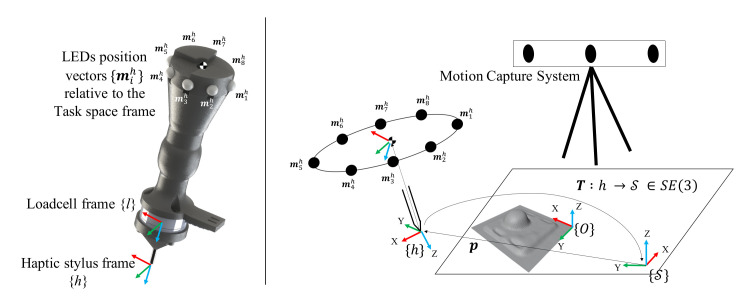
The haptic stylus is equipped with motion capture LED’s for kinematic data and a 6-axis loadcell (ATI Mini40) for forces data. The *SE*(3) filter provides an estimate for the position p and rotation R∈SO(3) of the haptic stylus frame *h*. An initial calibration must be done to express data obtained from haptic stylus frame (h→{S}) and the mesh obtained in object frame (O→{S}) in space frame. The forces sensed in the loadcell frame {l} were also expressed in {S}.

The proposed framework refines the mesh* using the haptic stylus position (p(t)) and force data (F(t)). Note that all these quantities must be expressed in the space frame {S}, i.e., the necessary transformations for the mesh variables ({O}→{S}) and the haptic stylus variables ({h}→{S}), must be computed between the different reference frames shown in Figure 13. The transformation from haptic stylus frame to space frame is:

(13) $$T=\left[\begin{array}{cc} R & p\\ 0 & 1 \end{array}\right]$$

where, R is the rotation matrix that describes the 3D orientation of the haptic stylus and p is the 3D position of the haptic stylus w.r.t space frame {S}. A similar transformation $T_{O}$ was calculated for the mesh variables by using the texture map obtained from the 3D scan. The space frame was visually located in the image space and the transformation matrix was constructed by using calibration points manually selected in *Blender*, an open source rendering engine used for the purposes of visualisation in this work.

The geometric refinement is performed in the neighborhood of the closest face $f^{*}$ with barycenter $b^{*}$ to p in mesh*. In this work, the closest point after a manual initialisation, was iteratively searched for in real-time within all the faces *f* in the 1-neighborhood *ROI* as discussed in Equation (12) in Section 4.3.

The normal $n^{*}$ for this f* is obtained from the mesh structure. Since the loadcell frame {*l*} is aligned with the stylus frame {*h*}, the *actual* haptic feedback ${\left[\begin{array}{ccc} f_{x} & f_{y} & f_{z} \end{array}\right]}^{T}$ sensed in {*l*} was converted into space frame coordinates by a simple rotation transformation as:

(14) $$F=R{\left[\begin{array}{ccc} f_{x} & f_{y} & f_{z} \end{array}\right]}^{T}$$

The *hatptic update* of f* is then performed as per the Equations (6) and (7) in Section 3.4 and a Laplacian deformation of the 1-neighborhood *ROI* was used to refine the immediate locality as discussed in Section 4.3. We next present the experimental details and the geometric refinement of features via haptic exploration of features and the consequent results.

### 5.2. Experimental Protocol

**Figure 14:**
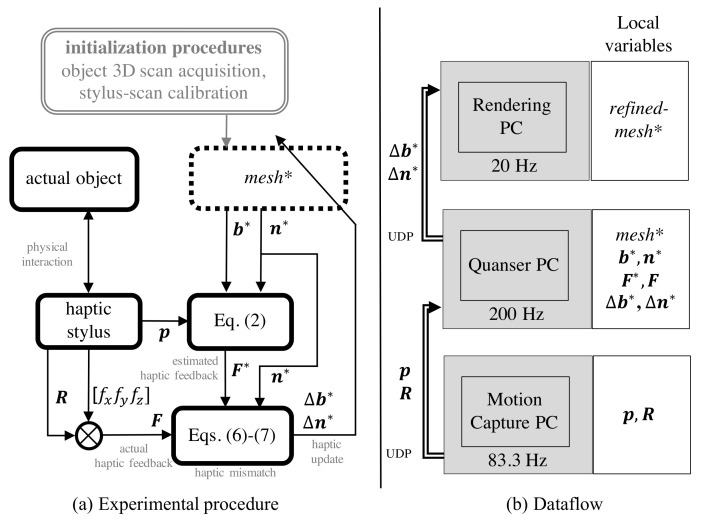
Procedure of geometric refinement of mesh* through visual inspection by the human operator.

The procedure followed to perform the geometric refinement of features is explained in Figure 14a. The human in the loop allows for *visual inspection* of the features deemed important to the process and enables interactive usage of the proposed framework. The 3D scan is updated based on the perceived haptic mismatch until it is minimised. The 3D scans of the two objects with more than 4 holes missing, and smoothed out edges and slots were refined using the proposed framework. All the missing features were successfully refined and feature detection was performed in post-processing.

The experiment was conducted using three Desktop machines (see Figure 14b): Quanser PC, Motion Capture PC and the Rendering PC. The Quanser PC was connected to a *Quanser QPIDe Terminal Board* and analog data from the *ATI Mini40* 6-axis F/T sensor (mounted between the handle and the pen-tip—see Figure 13) was sampled at 200 Hz. The *ATI Mini40* 6-axis F/T loadcell outputs the sensed forces in terms of analog voltages for: *f_x_*, *f_y_* and *f_z_*. These readings were calibrated to reflect the sensed forces (in *N*) in the loadcell frame of reference {*l*} (which is aligned with {*h*} at all times). Note that for calculating the haptic mismatch, these forces must be converted and expressed in space frame {S}. We next discuss the process of obtaining the pose of the haptic stylus, which allows for this {h}→{S} conversion of the sensed forces.

The Motion Capture PC communicated with the *PTI Phoenix Visualeyez II VZ4000V* Motion Capture system to track the 3D position of Motion Capture LEDs (markers) mounted on the hand-held haptic stylus. The haptic stylus pose was calculated from this and a UDP socket was written to communicate this information (p(t)) to the Quanser PC.

The pose of the haptic stylus was computed by implementing an iterative filter that considers the pose of a rigid body in space and describes it as a spring-damper problem in linear and rotational terms. Briefly, this filter makes use of the redundancy in the number of markers mounted on the object to provide a real-time pose of the haptic stylus, despite occluded markers. Ultimately, for the working of the proposed framework, the pose of the haptic stylus is needed. For more details, the reader is referred to [51] The rotation R obtained from the pose is used to compute the forces sensed by the loadcell in space frame, which is the actual force feedback F to be used for refining the geometric features in the proposed framework.

**Figure 15:**
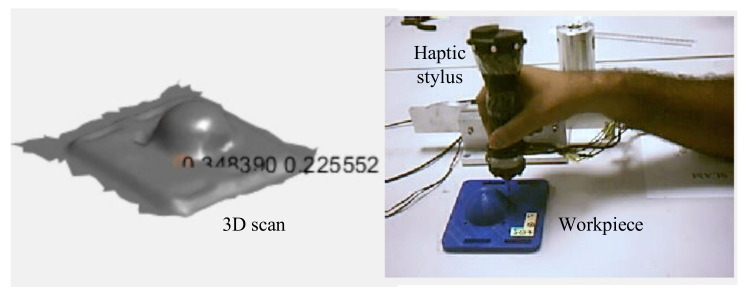
Interactive refinement (see uploaded video [52]) of the geometric features along the path of haptic exploration—in this case, the edges and holes of the object.

All of this information was combined on the Quanser PC to compute the *haptic update* and morph mesh* to conform to the actual surface geometry. To decrease the burden of computation, the rendering was done on another PC, labelled as the Rendering PC in Figure 14b. The vertex set of the morphed neighbourhoods were communicated through UDP to the Rendering PC to help visualize the result of the *haptic update*. A snapshot from the video of real-time haptic manipulation is shown in Figure 15.

The haptic stylus was used by a human operator to perform haptic exploration to refine the missing features in a 3D scan of an object. Since F is an actual force feedback during the experiments, only the *expected* interaction force $F^{*}$ was calculated using haptic stylus position p(t) and the refinement of mesh* was done. The interactive nature of the proposed framework may be observed in the compiled videos of haptic exploration [52].

### 5.3. Geometric Refinement via Haptic Exploration

**Figure 16:**
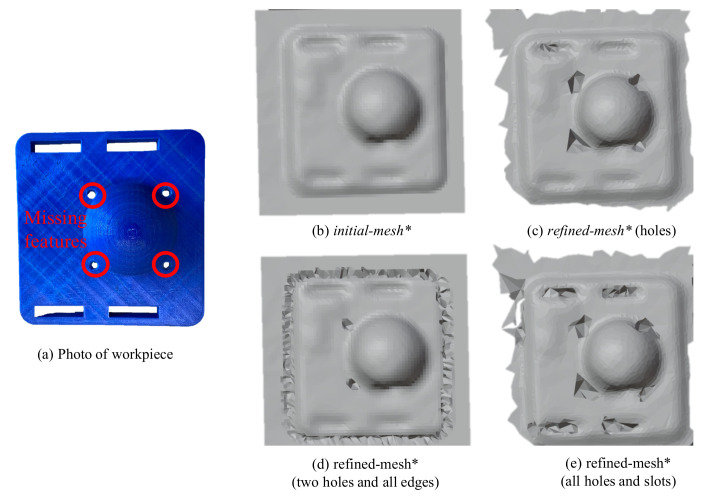
The haptic stylus was used to refine the features marked on the actual object. The segmented portion of the 3D scan highlighting the difference after haptic adaptation is shown here.

**Figure 17:**
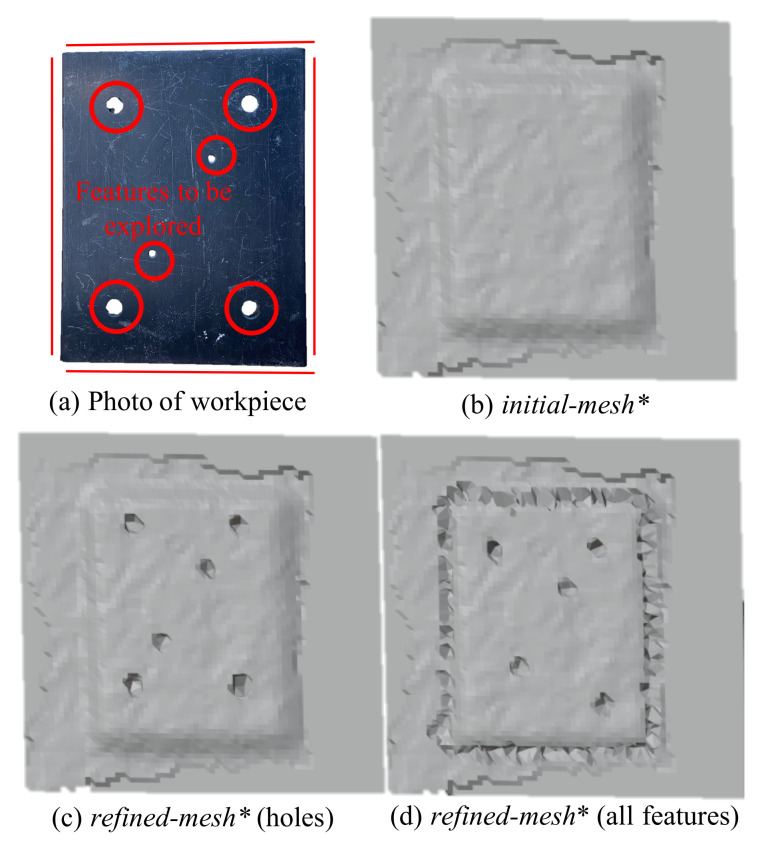
(**a**) The object of interest, (**b**) its initial-mesh* and (**c**,**d**) refined-mesh*.

We now discuss the results of selective feature refinement in two different objects. Specifically we work with holes, edges and slots as seen in Figure 16 and Figure 17. The viscous coefficients of haptic update, i.e., α = 0.1 mN${}^{-1}$s${}^{-1}$, β = 0.1 N${}^{-1}$s${}^{-1}$ for state dynamics’ evolution were heuristically set so that mesh* responded intuitively to the contact force imposed by the human operator. The rule of thumb to set these parameters is to observe the update rate of the mesh which depends on the sampling rate of position and haptic feedback and the stability of the mesh update. It is advisable to initiate at a very low value—which makes the update very sluggish and then increase until it is responsive enough to human movements. Upon performing haptic exploration on features marked in red in Figure 16, the refined mesh* clearly showed features absent in the initial mesh*. It may be noted that the framework allows for selective refinement as seen in the different results, as opposed to an end-to-end highly accurate solution that expensive 3D scanners offer. This allows for the framework to be used flexibly and is also useful in defining the scope for feature recognition later. In Figure 16c, only the holes of the object were refined, in (d) the edges were isolated and (d) shows the holes and slots highlighted better. We next present a methodology to benchmark our experimental results numerically.

**Figure 18:**
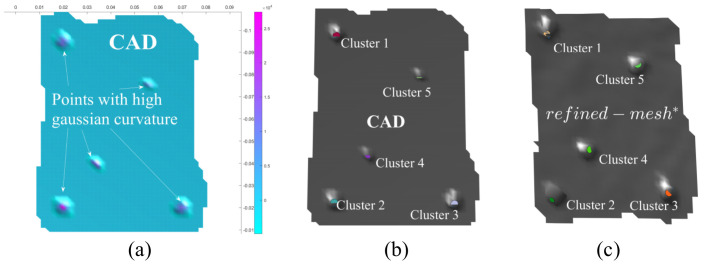
(**a**) Gaussian Curvature evaluated on the region of interest in the CAD of the object—Points in purple indicate depressions that help identify features like holes in this case, (**b**,**c**) Clustering based on gaussian curvature to identify and locate features like holes in this case.

The objective of the proposed framework is to enable localisation despite the poor quality of the 3D scans obtained from inexpensive scanners. To numerically validate the effectiveness of the proposed framework, we attempted to localise and match the *refined* geometric features using iterative closest point algorithm. Specifically, the refined-mesh* of the object in Figure 17a and its CAD (Figure 18a) were compared using this method. The procedure used was as follows: (i) Obtaining of *initial-* mesh* (Figure 17b) of object (Figure 17a), (ii) Performing haptic exploration over *all* features of interest (Figure 17d), (iii) Gaussian curvature evaluation on the meshes for this *refined-* mesh* and CAD, (iv) Clustering over the meshes to obtain *control points*, (v) Point set registration on the *control points* to align the refined-mesh* and CAD-mesh (Figure 18a) and (vi) After converting the meshes to point clouds, a cloud to cloud comparison to evaluate the error before and after haptic exploration. Note that haptic exploration was all the features to be highlighted in the object, i.e., holes and edges. The Gaussian Curvature [53] with a heuristic threshold of 0.3 was evaluated over the segmented region of interest in CAD (Figure 18a) and the refined-mesh*.

Following the evaluation of gaussian curvature on both CAD mesh and refined mesh, we now have a set of vertices per mesh which marked a high curvature. k-means clustering was performed on these vertex sets to derive centroids of the features with k=5 (may be automated depending on application). These clusters may be seen in Figure 18b,c. These cluster centroids are vital to perform mesh alignment before comparing the two meshes for geometric differences. Note that without *haptic update* performed over the initial-mesh*, obtaining these features or localizing these clusters would not have been possible.

**Figure 19:**
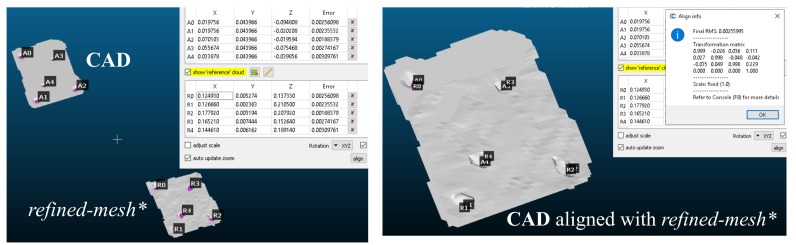
To perform a comparison between meshes, they need to be first aligned to each other. This was done by point set registration (in *CloudCompare*) with control points defined as the the clusters obtained from Gaussian curvature evaluation over both the CAD and refined-mesh*.

Point set registration was performed to align the two meshes (i) initial-mesh* and (ii) refined-mesh* with CAD as shown in Figure 19. The control points derived from the gaussian curvature evaluation play a key role in performing this alignment of meshes—both for initial-mesh* and refined-mesh*.

**Figure 20:**
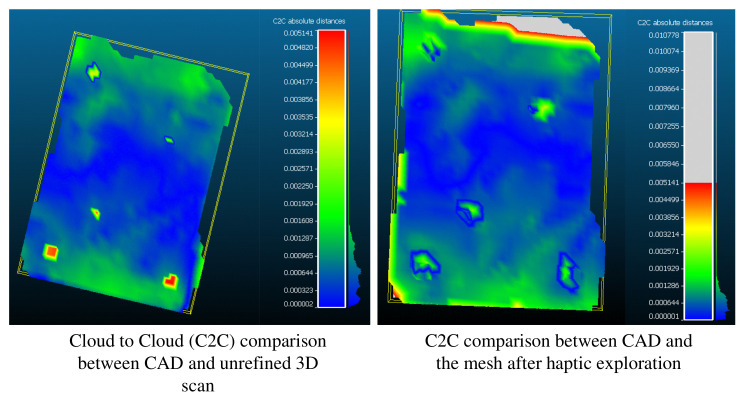
The CAD of the object with the features of interest was compared with the (i) unrefined 3D scan of the object and (ii) the updated 3D scan after haptic exploration. For the same C2C absolute distance range, it may be observed that the haptic refined scan has lower error. The key contribution of this framework therefore is to localize features that are absent in a 3D scan using haptic exploration.

Post alignment, the final step of evaluating the performance of our framework is to check if the geometric differences between the CAD and the refined scan were indeed less than those with unrefined scan. It may be observed in Figure 20 that the cloud to cloud (C2C) absolute distances at the holes (which were the features that we performed haptic exploration over) are indeed lower for the refined-mesh*.

Although the error does not vanish owing to human error, calibration error, etc. in the experiments shown above, the ability to perform haptic exploration of the workspace and update the robot’s knowledge is of critical importance in applications where the robot needs to physically interact with features like holes, edges, corners that may be absent in the 3D scan of the environment.

## 6. Conclusions and Future Scope

The proposed framework is a pre-manufacturing step for an operator to refine missing geometric features in a 3D scan before automated manufacturing can take place. The novelty of this framework is to use *actual* haptic feedback to perform virtual sculpting. The idea of *haptic mismatch* was introduced and defined as the error between the *actual* haptic feedback and the *expected* haptic feedback. The *expected* haptic feedback is computed using the geometry available from the 3D scan and the goal of the framework is to refine the 3D scan by physically interacting with the actual object via the haptic stylus to minimize *haptic mismatch*. The proposed framework was experimentally validated. In particular, an object was scanned and the haptic stylus was used to refine features like holes and slots which were missing in the initial 3D scan. All the missing features were successfully refined in the 3D scan using the framework.

Deploying cost-effective robotic systems in low volume high mix contexts, would face challenges in 3D perception. The significance of this work is in bridging this gap by providing a framework capable of harnessing position and force feedback to improve 3D perception and make automation more accessible. One of the limitations of our work is that the mesh updating does not keep memory of *where* it was updated, this is left entirely to the operator (who visually assesses the updating of the geometry). As part of future work, one possibility is to introduce this memory in the form of texturing or other dynamic properties of the mesh, e.g., so that previously updated areas are made less malleable. Other limitations include sensitivity to noise and position calibration of haptic stylus to scan. This can be also addressed in the future with a probabilistic version of this framework. The comparability of the tool shape with the size and shape of the targeted feature for refinement is important for effective usage of this framework. Additionally, the surface friction will also play a role in certain tasks—in this work we do not include this as a property of the mesh.

## Figures and Tables

**Table 1:** List of variables for *Haptic Update*.

Symbol	Variable Name
p	Haptic stylus position
$b^{*}$	Estimate of the closest point on surface
$n^{*}$	Estimate of the surface normal
F	*Actual* Haptic feedback
$F^{*}$	*Expected* Haptic feedback
*h*	Normal distance between p and scan
α	Linear admittance of face dynamics
β	Rotational admittance of face dynamics
δ	Haptic rendering inflection point
